# Assessment of Good Practices in Community-Based Interventions for Physical Activity Promotion: Development of a User-Friendly Tool

**DOI:** 10.3390/ijerph18094734

**Published:** 2021-04-29

**Authors:** Sofia Franco, Cristina Godinho, Catarina Santos Silva, Bruno Avelar-Rosa, Rute Santos, Romeu Mendes, Marlene Nunes Silva

**Affiliations:** 1 Programa Nacional para a Promoção da Atividade Física, Direção-Geral da Saúde, 1049 Lisboa, Portugal; godinhocristina@gmail.com (C.G.); catarinasantosilva@dgs.min-saude.pt (C.S.S.); bruno.ibe@gmail.com (B.A.-R.); rsantos.ciafel@fade.up.pt (R.S.); romeuduartemendes@gmail.com (R.M.); marlenesilva@dgs.min-saude.pt (M.N.S.); 2 Faculdade de Motricidade Humana, Universidade de Lisboa, 1495 Lisboa, Portugal; 3 Católica Research Centre for Psychological, Family and Social Wellbeing, Universidade Católica Portuguesa, 1649 Lisbon, Portugal; 4 CIPER, Faculdade de Motricidade Humana, Universidade de Lisboa, 1495 Lisboa, Portugal; 5 Faculty of Sports Science and Physical Education, University of Coimbra, 3040 Coimbra, Portugal; 6 Research Group on Culture and Education, University of Girona, 17004 Girona, Spain; 7 Qantara Sports, Dubai 122560, United Arab Emirates; 8 Research Centre in Physical Activity, Health and Leisure, Faculty of Sport, University do Porto, 4200 Porto, Portugal; 9 Laboratory for Integrative and Translational Research in Population Health (ITR), 4050 Porto, Portugal; 10 EPIUnit-Instituto de Saúde Pública, Universidade do Porto, 4050 Porto, Portugal; 11 Northern Region Health Administration, 4000 Porto, Portugal; 12 CIDEFES, Faculdade de Educação Física e Desporto, Universidade Lusófona de Humanidades e Tecnologias, 1749 Lisboa, Portugal

**Keywords:** health promotion, physical activity programs, municipalities, evaluation tool

## Abstract

Tools to identify good practices in the design, implementation, and evaluation of physical activity community-based interventions (PACIs) are key to address the physical inactivity pandemic. Existing tools tend to be extensive and with limited applicability to assess small-scale PACIs. This work aimed to report the development and preliminary validity results of a simple, practical, and user-friendly tool to evaluate PACIs in local/municipal contexts. Eighty-six good practice characteristics defined by the World Health Organization (WHO), the Joint Action Framework on Chronic Diseases (CHRODIS), and an umbrella review of good practice characteristics of diet and physical activity interventions were initially extracted and refined in four rounds of revision from an expert panel using a Delphi-type methodology and rated on their relative importance. A pilot application was conducted, and data on the tool usability and applicability were collected through three semi-structured interviews with specialists and coordinators of local/municipal PACIs. For preliminary validation, the refined tool was applied to five community-based programs mostly aimed at an elderly population. The final tool included thirty-four selected characteristics, with a brief explanation and practical examples for each, under three main sections: design, evaluation, and implementation. Each characteristic has a rating (i.e., somewhat important, highly important, mandatory) and a percentage weight. Preliminary validation of this tool pointed to an adequate evaluation of good practice characteristics of municipal PACIs in a reliable, practical, and user-friendly way. Given its adequacy, this tool can support the definition of quality standards for PACIs, encouraging their dissemination and adoption at a regional or national level.

## 1. Introduction

Despite the growing evidence highlighting the health-related benefits of regular physical activity (PA) [1,2], the sustained adherence to this fundamental health determinant is still far from being achieved at a population level. Physical inactivity, defined as an activity level insufficient to meet the current physical activity recommendations [3], is considered a worldwide pandemic, with inactivity levels achieving about 70% in some countries [4]. Worldwide, it is estimated that physical inactivity is responsible for 6% to 10% of major non-communicable diseases (e.g., coronary heart disease, type 2 diabetes, and some types of cancer) [3].

One of the simplest and most effective ways to promote PA is the development and implementation of physical activity community-based interventions (PACIs) and initiatives [5,6]. The World Health Organization (WHO), in its Global Action Plan on Physical Activity 2018–2030 [7], highlighted the crucial role of PACIs in tackling physical inactivity worldwide, especially when these use a whole-of-system approach. Municipalities have a key role in mobilizing local resources, being in direct contact with the local population, sports clubs, and entities, with a depth of knowledge of the local context where these interventions are implemented [6,8,9].

Despite their advantages, community-based interventions promoted by municipalities have some limitations; for example, a poor design and a lack of proper assessment can hamper their validity and effectiveness in promoting PA [10,11]. Indeed, a large part of community-based interventions has conceptual design insufficiencies, as well as insufficiencies in the reporting of the methods used in the intervention [12]. In this regard, the evaluation and validation of these kinds of interventions would benefit from an evaluation tool to ensure compliance with recognizable good practice characteristics for promoting PA. Such tool would also be useful for the planning and developmental phases of the intervention [8,13], contributing to addressing the WHO recommendation for the development of effective community actions, based on scientific evidence and supported by validated evaluation systems [7,14].

Good practices are characteristics of community-based initiatives or interventions, implemented in real-life settings, tailored to their context of implementation, and accepted by the target population and stakeholders, that have shown to be effective, produce good results, efficient, sustainable, replicable, and ethical [15,16]. Indeed, a good practice is not only a practice that is good but a practice that has been proven to work well and produce the desired results and is therefore recommended as a model. It is a successful experience that has been tested and validated, in the broad sense, and has been repeated and deserves to be shared so that a greater number of people can adopt it [15].

Some systems and tools for the evaluation of good practice characteristics in community-based interventions have been defined by international entities, literature reviews, and conceptual frameworks [15,16,17,18]. This set of characteristics enables the description and evaluation of PACIs, allowing the identification, recognition, and dissemination of quality-implemented community-based interventions, according to the various dimensions of good practice characteristics. These may also help in the definition of quality standards for PACIs, encouraging their dissemination and adoption at a regional or national level.

Notwithstanding their value and contribution to the dissemination and validation of good practices, the currently available evaluation tools and systems are not user friendly, presenting some limitations when applied to community-based contexts. For example, in the evaluation of small-scale community-based interventions, such as municipal/local initiatives, many of these tools are extensive, have reduced practical applicability, and do not take into account the real constraints of small-scale community-based interventions, contributing to difficulties in its evaluation [19].

To fill this gap, there is a need to develop a simpler tool that is easy to apply and suitable to municipal/local contexts, capable of gathering relevant information on existing interventions in a systematic way, enabling and fostering the development, identification, evaluation, and dissemination of good practice characteristics in PACIs. The aim of this study was to report the development and preliminary validity results of a simple, practical, and user-friendly tool, to evaluate PACIs in local/municipal contexts.

## 2. Methods

### 2.1. Development of the Tool

For the development of the tool, the criteria for the Good Practice Appraisal Tool for obesity prevention, defined by the WHO [18], the Joint Action Framework on Chronic Diseases (CHRODIS) work package on good practices in health promotion [15], and an umbrella review of good practice characteristics of diet and physical activity interventions [17] were considered, as they are reference documents for the definition of good practices in health and PA promotion.

To reduce and simplify the good practice characteristics identified, a panel of five experts was consulted. These experts have PhDs or Master’s degrees in exercise and health and a good track record in developing and evaluating PACIs.

Through a Delphi-type methodology [20], using four rounds of revisions, the list of characteristics was refined.

I. In the first round, the main goal was to reach a consensus on the best term and description used for each good practice characteristic, given that, in some cases, different names were used to define the same characteristic, and sometimes the same characteristic was defined differently.
II. Then, with this new list of good practice characteristics, the expert panel was asked to rate each characteristic based on their relative importance on a scale of 1 to 10.
III. In the third round, all the ratings were grouped, and a discussion was conducted to reach a consensus among the expert panel on the rating for each one of the good practice characteristics listed previously by recoding the previous answers ranging from 1 to 10. Then, a final rating on the relative importance for each characteristic was established, where each characteristic was rated with 1 (i.e., somewhat important), 2 (i.e., highly important), or 3 (i.e., mandatory). This rating was also converted into a percentage score, from 0 to 100%, accordingly.
IV. Finally, in the fourth round of revision, small edits were performed, and a final tool, with a list of the good practice characteristics and respective ratings, was approved.

### 2.2. Pilot Application and Preliminary Validation of the Tool

A pilot application was conducted to collect information on the tool’s usability and applicability. For this, three semi-structured interviews with relevant stakeholders were conducted. The group of relevant stakeholders comprised specialists in implementing and evaluating PACIs who hold PhDs in exercise/health sciences and coordinate community-based interventions promoting PA (with extensive experience in developing, implementing, and evaluating community-based interventions, as well as a scientific background in behavior change techniques for health promotion, specifically in physical activity). These interventions were all derived from research-based initiatives, implemented in community settings, according to well-defined and standardized procedures in relation to both intervention contents and assessments. All the selected interventions were delivered by a team of exercise and health-trained specialists and encompass over 400 participants each.

Each interview was recorded and divided into four parts: (1) presentation of the study aim and background, (2) description of the procedure followed for the tool development, (3) application of the tool to the PACI coordinated by the interviewee, and (4) questions and feedback. The tool was then refined based on the comments that emerged from these key interviews. For concurrent validity, the interventions chosen were PACIs whose quality in terms of good practices had already been attested by macro reference frameworks (e.g., the European Union and the Intersectoral Commission for the Promotion of Physical Activity in Portugal (CIPAF) [21]).

Then, for preliminary validation, the tool was applied to five community-based interventions promoting PA in Portugal. To ensure national representativeness, one community-based intervention was chosen from each one of the five regions of Portugal’s mainland (North, Centre, Lisbon Metropolitan Area, Alentejo, and Algarve). The community-based interventions were chosen according to their implementation scale (i.e., a greater number of participants), ranging between 200 and 2000+ participants. To gather information on these interventions, the city council and the interventions’ websites were initially consulted. As the information available online proved to be insufficient, a telephone interview was conducted with the coordinators of the interventions to complete the tool application. The coordinators provided information related to all good practice characteristics listed and a score was given to each intervention. Each telephone interview lasted about 30 min.

## 3. Results

### 3.1. Development of the Tool

**Figure 1:**
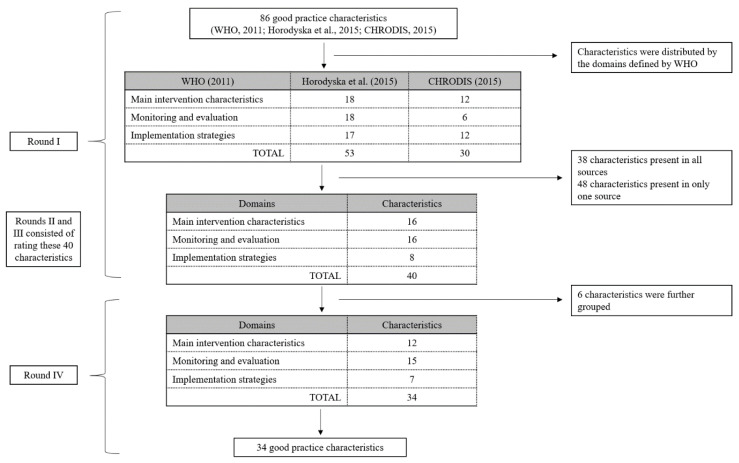
Process of developing the tool for evaluating good practice characteristics in PACIs.

Based on the existing good practice characteristics listed in the three references aforementioned, 86 characteristics were identified. To simplify the analyses, the 53 characteristics defined by the umbrella review [17] and the 30 criteria defined by CHRODIS [15] were distributed by the three main domains defined by the WHO [18] (see Figure 1). A total of 38 characteristics were considered redundant, as they were mentioned in all key documents (e.g., “Target audience well defined” [17] and “Target population/s are defined” [15]), while the other 48 characteristics were present in only one of the sources (e.g., “General use of behavioral change techniques” [17]). Most of the characteristics identified were described using different wording and terminology across the different sources (e.g., “Reach—the strategy is likely to involve a large percentage of the target population” [17] vs. “Empowerment and Participation—the intervention achieves meaningful participation among the intended target population” [15]).

Data from the Delphi-type methodology showed the following (see Figure 1):

I. In the first round of revision by the expert panel, the initial list of 86 characteristics was reduced to 40, after the elimination of duplicates, and distributed by the three main domains (16 main intervention characteristics, 16 monitoring and evaluation characteristics, and 8 implementation strategies).
II. In the second round of the Delphi process, the expert panel rated each characteristic on a scale from 1 to 10 based on their relative importance. At this point consensus was reached for 11 out of the 40 characteristics regarding their relative importance; all other characteristics were discussed among the expert panel until a consensus was reached.
III. Following this debate, in the third round of revision, it was considered that a rating scale ranging from 1 to 3 was more appropriate and easier to apply. Therefore, each characteristic was rated, based on their importance, with 1 (i.e., somewhat important), 2 (i.e., highly important), or 3 (i.e., mandatory). Seven characteristics were rated with 1, eight with 2, and the remaining with 3. This rating was also converted into a percentage score. From this process, the list created now included a specific rating, as well as a percentage ratio, for each good practice characteristic.
IV. Finally, in the fourth round, small edits were made to the list, six characteristics were further grouped, and the final tool consisted of 34 good practice characteristics.

### 3.2. Pilot Application and Preliminary Validation of the Tool

From the pilot study of the tool, the experts interviewed considered that the development of the tool was adequate for the context of its application and the rating attributed to each characteristic was proportionate. Each application of the tool lasted around 30 min, which was also considered adequate, and the interventions assessed were ranked high on a percentage rate (between 96% and 100%), as expected due to concurrent validity testing efforts, which confirms the adequacy of the tool.

However, respondents of the key interviews suggested some refinements to the tool to make the application process easier. It was suggested that each characteristic could be transformed into a question (instead of an item-style application), supported by practical examples and definitions for some of the most abstract or difficult to understand characteristics (e.g., comprehensive approach to health promotion, estimation of effect sizes, etc.) (see Supplementary Materials). Remarks were also made concerning the need of adapting the “follow-up evaluation” as a requirement. Indeed, as some interventions operate all year, every year, cyclically with the same group of participants, a continuous approach to this evaluation process, instead of a follow-up approach, was considered to be more adequate. These changes were made to the tool, and a new version was created (see Table 1).

Following this process, the preliminary validation of the tool was carried out. Results showed that all interventions scored highly regarding good practice characteristics. Scores ranged between 78.0% and 94.7%. The mean score was 88.3%.

In the main intervention characteristics, the scores were between 44.0% and 55.5% (three interventions met all the characteristics). The mean score was 52.2%. Four out of five interventions targeted older adults (55+ years) and seniors, and one was aimed at adults over 40 years. They all included weekly exercise sessions complemented with other activities (e.g., seminars pertaining to other health-related themes, community events, social gatherings, etc.), were evidence based, provided a consent form, developed a manual/protocol script, and specified the qualifications of the practitioners involved (exercise physiologists, mainly). Three out of the five reported using behavioral change techniques to reach their objectives (e.g., self-monitoring).

Regarding the monitoring and evaluation processes, the lowest score was 16.5%, and the highest was 20.2%. The mean score was 18.8%. None of the interventions responded fully to this domain. However, all the interventions calculated the total costs of the interventions, on an annual basis, and monitored participation rates and drop-outs, described the methods used for the process and result evaluation, and the recruitment strategy used. Nonetheless, cost-effectiveness analyses, cost per participant, or effect sizes were not calculated by any of the PACIs considered, and only three interventions evaluated participants’ satisfaction.

Regarding the implementation strategies, the scores ranged between 14.8% and 19.0%, and the mean score was 17.4%, so none of the interventions were able to score the full 20.0% for this domain. All the interventions reported on the resources needed, tried to integrate resources already available and seek to establish partnerships with relevant organizations or stakeholders, ensuring intervention sustainability. Only three interventions offered specific training for the practitioners, regarding aspects of implementation. The transferability of the intervention was not evaluated by any of the PACIs.

## 4. Discussion

This study reported the development and preliminary validity of a user-friendly tool to evaluate the good practice characteristics of PACIs. According to stakeholders’ feedback on the easiness of completion, reinforced by the five interviews on the preliminary application, the refined tool has shown to be easy to understand and to fill in, i.e., user friendly.

From an initial list of 86 good practice characteristics, it was possible to systematize data and eliminate redundancies into a final tool of 34 good practice characteristics. Having a short and simpler-to-use tool, containing illustrative examples for each domain assessed, may foster its adoption by different agents managing PACIs, such as municipalities and public health stakeholders, as well as practitioners and researchers working in local communities [22]. Additionally, besides simplifying the previously identified characteristics, the tool focuses on establishing well-defined and easily identifiable good practice characteristics, and not only on broad categories that may be difficult to apply in real contexts [23].

Following the pilot application of the tool on experts and coordinators of PACIs, it was possible to obtain a refined version of the tool, with more clarified key terminology, and illustrating examples for each characteristic, facilitating its comprehension and completion. The refined tool was also considered valid (concurrent validity), rating the interventions evaluated during this phase with high scores, as expected. In the preliminary validation of the tool, through its application to five local/municipal PACIs from the five regions of Portugal, it was found that the tool is simple to apply, easy to understand, and that its application takes approximately 30 min, which is considered adequate.

Regarding the good practice characteristics listed in the tool, the results highlighted its relevance and applicability, with a few exceptions. None of the PACIs assessed through the tool fulfilled some of the included characteristics: (i) report of financial cost per participant, (ii) report of financial costs related to the health benefits obtained—cost-effectiveness analysis, (iii) the estimation and report of effect sizes, and (iv) the assessment of the intervention’s transferability potential to other populations/contexts. This finding is indicative of a generalized weakness in PACIs that needs to be further addressed. Indeed, the need to specify the financial cost per participant was highlighted in an umbrella review [17] as a good practice characteristic to be considered. Although all the interviewed coordinators reported the annual financial costs that are required to deliver the intervention, cost per participant was not calculated. However, this could be easily done by dividing the total annual financial costs of the intervention by the total number of participants, which can be assessed through the reporting of the intervention’s participation and dropout rates. In this sense, in future applications of this tool, it is plausible to consider this characteristic as fully achieved if the intervention does assess and report the annual financial costs and participation rate.

The assessment and report of financial costs related to the health benefits obtained (cost-effectiveness analysis) were also defined as a good practice in the same umbrella review [17]. Cost-effectiveness analysis aims to compare the costs of an intervention with its effect/outcome [24]. This type of analysis is relevant because it helps identify which interventions have the potential to produce the best health outcomes for their participants, using the least resources, which is particularly useful for intervention comparisons set out to achieve the same objectives [25]. Although it is considered a good practice characteristic, none of the PACIs evaluated reported it. PACI cost-effectiveness analyses may be difficult to estimate, probably because the entities that implement these interventions (i.e., municipal councils) may not have the necessary resources or knowledge/training to carry out this type of analysis. To overcome such potential barriers, the existence of partnerships between municipalities and relevant stakeholders (e.g., universities, government bodies) could be advantageous [26].

Another good practice characteristic that was not assessed in any of the interventions was the estimation and reporting of effect sizes. This measure compares the magnitude of the difference in the results obtained in different groups or different moments of evaluation of an intervention, identifying for which group the intervention was more effective or if the intervention was effective at all [27]. Effect sizes may not have been estimated by these interventions for two reasons. First, in the evaluated interventions, all groups of participants are subjected to the same intervention and there was no control group for comparison of effects. Second, municipal councils may not have the resources nor the knowledge/training necessary to carry out this type of analysis. As suggested above, the establishment of partnerships in this regard may be beneficial [27].

The transferability potential of the interventions to other populations or contexts was also not assessed, at least entirely. This transferability and adaptability of an intervention are important to develop a process of dissemination to other communities of the most effective interventions to ensure the promotion of PA at a larger scale [8]. Some of the interventions’ coordinators who were interviewed mentioned that the design of the intervention was based on an existing PACI that had been implemented elsewhere in the country and that, therefore, they were contributing, in a way, to the dissemination of those interventions. However, no formal study had been carried out on the transferability potential of the intervention.

The developed tool includes some evaluation characteristics that may be too inaccessible for this type of PACIs (e.g., cost-effectiveness analysis, estimation of effect sizes). Despite this, it may be useful for these characteristics to remain in the tool, given one of its intentions is to allow for self-evaluation (i.e., by answering this set of questions, coordinators of PACIs can become aware of the areas and good practice characteristics not covered by the intervention). If the evaluation of PACIs through this tool highlights that an intervention does not meet some of the good practice characteristics, it may stimulate coordinators to refine and improve current practices in order to fulfill the missing characteristics. This pedagogical nature of the tool will help to improve the characteristics of existing PACIs and serve as an example and model to be followed by new interventions that are being developed.

It is important to underline that this study intended to develop a good practice evaluation tool, which has been subjected, so far, to a preliminary validation. Hence, despite its strengths, some limitations should be acknowledged. For example, the selection process of PACIs for the preliminary testing of the tool dictated a low heterogeneity in the evaluated interventions, all of them being of good quality and meeting most of the proposed characteristics (all with scores above 70%). This factor may have contributed to lower quality or small-scale interventions being left out of the evaluation process. Therefore, this tool needs to be further validated with more heterogeneous PACIs. Additionally, the small number of PACIs that have been evaluated in the validation process prevents us from generalizing the obtained results and comparing the assessed interventions according to specific features, such as, for example, the effect of regional characteristics in the results. This is, thus, an important avenue for future research using this tool.

To disseminate its usage, in the future, this tool can be adapted into an online data collection form (e.g., online questionnaire). This will enable coordinators of PACIs to independently submit their interventions for assessment of good practices, with the possibility of instantly receiving a score and further feedback regarding it. This online mechanism would serve to evaluate the interventions and enable the creation of a comprehensive database on existing municipal/local PACIs at a national level. Such a database would foster recognition and dissemination at the national level of good practice PACIs, which is in line with international recommendations [14]. As an example, it aligns with one of the operational goals set by the National Program for Physical Activity Promotion of the Portuguese Directorate-General of Health [28], specifically related to “monitoring and good practices: to promote the surveillance of PA determinants and the identification and enhancement of good practices in the promotion of PA and active aging in the community” [28]. The large-scale adoption of this tool could foster its clinical relevance [7] by supporting and elevating the standards in the development and evaluation of PACIs, some of which target populations with chronic disease, indirectly promoting their effectiveness and cost-effectiveness.

Moreover, although the applicability of the developed tool has only been validated in PACIs, this tool has the potential to be adopted in other areas of health promotion (e.g., community-based interventions promoting healthy eating habits, etc.) and in other countries. The defined good practice characteristics are not restricted to the area of PA but to health promotion in general, allowing this tool to be adapted and validated in a more universal way for community-based health promotion interventions in different contexts and settings.

## 5. Conclusions

The tool that was developed to assess good practices in municipal/local PACIs was found to be practical and adjusted to the community context, allowing the proper evaluation of good practice characteristics in these interventions. The tool presented herein has shown to be a user-friendly, low resource-demanding assessment of good practice criteria, including examples for each item that facilitate its use. Thus, it seems to be adequate to identify and evaluate good practices in local/municipal PACIs. This identification of good practices has the potential to help their dissemination on a regional or national scale. By being a more pragmatic tool and better adapted to the context in which community-based interventions take place, we expect it can contribute to foster the use of best practices in PACIs in the future.

## Figures and Tables

**Table 1:** Good Practice Characteristics Appraisal Tool.

Good Practice Characteristics Appraisal Tool			Percentage (Rate)
Main Intervention Characteristics (55.5%)			
Evidence/theory used in the development of the intervention is described			6.5% (3)
Target population is defined and justified (regarding risk and susceptibility factors)			6.5% (3)
Equity in access to intervention and throughout participation is ensured			6.5% (3)
Target behavior to change or to gain through participation is defined			6.5% (3)
Intervention has a comprehensive approach to health promotion, regarding individual, social and environmental factors			5% (2)
Detailed description of the intervention	Place, number, duration, and frequency of contacts with participants specified, as well as the total duration of the intervention		1.69% (3)
	Methods of content delivery are specified		1.69% (3)
	Themes/contents of each session are defined and justified		1.69% (3)
	Manual/protocol to support implementation exists		1.43% (2)
Behavioral change techniques are used, specified, and justified			5% (2)
Informed consent form, respecting ethical principles, exists			6.5% (3)
Qualifications and competences of the practitioners involved are specified			6.5% (3)
**Monitoring and Evaluation (24.5%)**			
Description of total financial costs of the intervention	Total financial costs of the intervention are specified, including cost per participant		3% (2)
	Costs in relation to obtained general health benefits are specified (cost-effectiveness analyses)		2% (1)
Description of evaluation framework	Process indicators/measures are specified		1.43% (3)
	Outcome indicators/measures are specified		1.43% (3)
	Methods used to measure outcomes are specified and justified		1.43% (3)
	All evaluation moments are specified		1.43% (3)
	Follow-up and/or sustained evaluation of participants is specified		0.78% (1)
Presentation of results and outcomes	Report of process evaluation results	Recruitment strategies and their reach are reported	1.43% (2)
		Participation and dropout rates throughout the intervention are reported	2.08% (3)
		Participant’s satisfaction rates are reported	1.43% (2)
		Unintended outcomes are monitored and reported	0.78% (1)
		Consistency/fidelity or changes made to the protocol are monitored	0.78% (1)
	Report of outcome evaluation results	Effect sizes are estimated and reported	1.3% (1)
		Evaluation results according to the stated goals and objectives are reported	2.9% (3)
		Negative consequences and outcomes are monitored and reported	2.3% (2)
**Implementation Strategies (20%)**			
Detailed description of the implementation strategies	Practitioners received training in aspects of implementation		1.43% (3)
	Human and material resources necessary for the implementation are specified		1.43% (3)
	Existing resources are used or integrated		1.43% (3)
	Organizational structures are clearly defined and described (e.g., work and communication flow, responsibilities)		0.78% (1)
Multidisciplinary and intersectoral partnerships for the development and implementation of the interventions are established			6.5% (3)
A long-term strategy for implementation is defined			5% (2)
Transferability of the intervention to other populations or contexts is assessed			2% (1)

## Data Availability

Data are available from the authors upon request.

## Supplementary Materials

The following are available online at https://www.mdpi.com/article/10.3390/ijerph18094734/s1, Doc S1: Good Practice Characteristics Appraisal Tool-Questionnaire.

## References

[1] World Health Organization (2020). WHO Guidelines on Physical Activity and Sedentary Behaviour.

[2] Pedersen B.K., Saltin B. (2015). Exercise as medicine—Evidence for prescribing exercise as therapy in 26 different chronic diseases. Scand. J. Med. Sci. Sports.

[3] Lee I.M., Shiroma E.J., Lobelo F., Puska P., Blair S.N., Katzmarzyk P.T. (2012). Effect of Physical Inactivity on Major Non-Communicable Diseases Worldwide: An Analysis of Burden of Disease and Life Expectancy. Lancet.

[4] Guthold R., Stevens G.A., Riley L.M., Bull F.C. (2018). Worldwide trends in insufficient physical activity from 2001 to 2016: A pooled analysis of 358 population-based surveys with 1·9 million participants. Lancet Glob. Health.

[5] Bauman A.E., Reis R.S., Sallis J.F., Wells J.C., Loos R.J., Martin B.W. (2012). Correlates of physical activity: Why are some people physically active and others not?. Lancet.

[6] International Society for Physical Activity and Health (ISPAH) (2020). ISPHA’s Eight Investments That Work for Physical Activity. www.ISPAH.org/Resources.

[7] World Health Organization (2018). More Active People for a Healthier World: Global Action Plan on Physical Activity 2018–2030.

[8] European Commission (2011). European Guide of Healthy Physical Activity and Sports Programmes. Methodology and Compilation of Best Practices.

[9] Misener K., Harman A., Doherty A. (2013). Understanding the local sports council as a mechanism for community sport development. Manag. Leis..

[10] Baker P.R.A., Francis D.P., Soares J., Weightman A.L., Foster C. (2015). Community wide interventions for increasing physical activity (Review). Cochrane Database Syst. Rev..

[11] Sallis J.F., Bull F., Guthold R., Heath G.W., Inoue S., Kelly P. (2016). Progress in physical activity over the Olympic quadrennium. Lancet.

[12] Hanson S., Jones A. (2017). Missed opportunities in the evaluation of public health interventions: A case study of physical activity programmes. BMC Public Health.

[13] Reis R.S., Salvo D., Ogilvie D., Lambert E.V., Goenka S., Brownson R.C. (2016). Scaling up physical activity interventions worldwide: Stepping up to larger and smarter approaches to get people moving. Lancet.

[14] World Health Organization (2013). Global Action Plan for the Prevention and Control of Noncommunicable Diseases: 2013–2020. http://apps.who.int/iris/bitstream/10665/94384/1/9789241506236_eng.pdf.

[15] Chrodis J.A. (2015). Joint Action on Chronic Diseases & Promoting Healthy Ageing across the Life Cycle—Work Package 5: Task 3. Good Practices in Health Promotion & Primary Prevention of Chronic Diseases. http://chrodis.eu/our-work/05-health-promotion/wp05-activities/selection/.

[16] JANPA (2015). Definition and Criteria of Good Practice for Childhood Obesity Prevention Programs in Kindergartens and Schools (WP6 Healthy Environments by Integrated Approaches). http://janpa-toolbox.eu/page.php?id=40.

[17] Horodyska K., Luszczynska A., Van Den Berg M., Hendriksen M., Roos G., De Bourdeaudhuij I. (2015). Good practice characteristics of diet and physical activity interventions and policies: An umbrella review. BMC Public Health.

[18] WHO Regional Office for Europe (2011). Good Practice Appraisal Tool for Obesity Prevention Programmes, Projects, Initiatives and Interventions.

[19] Ng E., De Colombani P. (2015). Framework for Selecting Best Practices in Public Health: A Systematic Literature Review. J. Public Health Res..

[20] Adler M., Ziglio E. (1996). Gazing into the Oracle: The Delphi Method and Its Application to Social Policy and Public Health.

[21] Diário da República (2017). Despacho n.o 3632/2017. 2a Série-No 83 de Abril de. https://dre.pt/home/-/dre/106943778/details/maximized.

[22] Green L.W., Glasgow R.E. (2006). Evaluating the Relevance, Generalization, and Applicability of Research: Issues in External Validation and Translation Methodology. Eval. Health Prof..

[23] Glasgow R.E., Emmons K.M. (2007). How Can We Increase Translation of Research into Practice? Types of Evidence Needed. Annu. Rev. Public Health.

[24] Levin H.M., McEwan P.J. (2000). Cost-Effectiveness Analysis: Methods and Applications.

[25] The Health and Environment Linkages Initiative (HELI) Cost-Effectiveness Analysis for Health Interventions. https://www.who.int/heli/economics/costeffanalysis/en/.

[26] Buse K., Mays N., Walt G. (2012). Making Health Policy.

[27] Sullivan G.M., Feinn R. (2012). Using Effect Size—Or Why the P Value Is Not Enough. J. Grad. Med. Educ..

[28] Programa Nacional para a Promoção da Atividade Física (2019). Programa Nacional para a Promoção da Atividade Física.

