# Supplementary material for: Assessment of Good Practices in Community-Based Interventions for Physical Activity Promotion: Development of a User-Friendly Tool
Source: Int J Environ Res Public Health. 2021 Apr 29;18(9):4734. doi: 10.3390/ijerph18094734 (PMC8124131; doi:10.3390/ijerph18094734)
Supplement: Supplementary file 1 [file ijerph-18-04734-s001.zip › ijerph-1187255-SI.pdf]

Assessment of Good Practices in Community-based Interventions for Physical Activity Promotion: Development of a user-friendly tool

Sofia Franco, Cristina Godinho, Catarina Santos Silva, Bruno Avelar Rosa, Rute Santos, Romeu Mendes, Marlene Nunes Silva

Supplementary Material

Good Practice Characteristics Appraisal Tool - Questionnaire

Questions about main intervention characteristics

1. Was the intervention based on any existing scientific evidence or theory?

Example: intervention was based on scientific evidence demonstrating the benefits of physical activity; intervention is based on self-determination theory

2. What is the target population for the intervention? Please justify this choice.

Example: intervention focused on promoting physical activity among men, as they are a population group usually discriminated in this type of intervention

3. Was equity in access to the intervention and throughout participation ensured?

Example: no one who was interested in the intervention and met the characteristics of the target population was denied participation

4. What behaviour/s is/are to be changed or acquired through participation in the intervention?

Example: increasing number of steps per day; reducing sedentary time by 1h a day; increasing number of days per week doing physical activity

5. Does the intervention present a comprehensive approach to health promotion, regarding individual, social and environmental factors?

Comprehensive health promotion approach - a strategy used for health promotion that develops individual competencies, creates supportive environments and positive relationships, and seeks to increase community involvement in these actions

Example: intervention includes group sessions that promote not only individual skills, but also the relationship with other participants and professionals involved, seeking to involve the whole community

6. How many, for how long and where are the sessions or contacts with the participants? What is the total duration of the intervention?

Example: intervention lasts for 3 months, with 12 sessions lasting 1h30 each, once a week, held in the local sports center

7. How are the contents or sessions of the intervention presented?

Example: in groups or individually; face-to-face or digitally

8. Which themes or contents are covered in each session or module of the intervention? Please justify this choice.

Example: 3 sessions are on the importance of physical activity and 3 sessions are on healthy eating, since the aim of the intervention is to promote an increased level of physical activity and to promote better food choices

9. Is there a manual or protocol to support implementation of the intervention?

Example: manual to support the practitioners implementing the sessions with a description of the intervention protocols

10. What is/are the behavioral change technique/s used in the intervention? Please justify this choice.

Behavioral change technique - active component of an intervention based on behavioral change

Example: goal setting strategies; giving feedback; ensure social support; use of a pedometer for self-monitoring the number of steps per day

11. An informed consent form was given to participants, prior to participation?

Example: clear description of intervention objectives, explanation of benefits and potential adverse effects was ensured

12. What qualifications or competences should the practitioners involved in the intervention have?

Example: degree in exercise and health; training in behavioral change techniques

Questions about monitoring and evaluation strategies

1. Have the total financial costs necessary to deliver the intervention been calculated, including the cost per participant? If so, please specify.

Example: 12500€ per year, with 120€ per year spent on each participant

2. Has a cost-effectiveness analysis been carried out? If so, please specify.

Cost-effectiveness analysis - economic analysis that compares the relative costs and outcomes/effects of one or more interventions and aims to minimize the costs of achieving a specific objective; it is expressed by the ratio of a measure of 'improved health' (e.g., years of life gained, days free of illness) to the costs associated with that measure

Example: how much did it cost to reduce the participants' systolic blood pressure by 10mmHg

3. What indicator/s or measure/s are used in the process evaluation?

Process indicators - factors that affect or reflect how an intervention was designed or implemented

Example: number of sessions given; barriers or enabling factors to intervention implementation

4. What indicator/s or measure/s are used in the outcome evaluation?

Outcome indicators - factors that indicate exactly what to measure, to understand whether the objectives/outcomes have been achieved

Example: participation increased number of steps per day by 1500 and reduced sedentary time by 60 minutes per week

5. Which evaluation method/s or instrument/s was/were used? Please justify this choice.

Example: pedometer; accelerometer; scale

6. How many assessment/evaluation moments existed? Please specify when these took place.

Example: 3 evaluation moments - at the beginning, middle and end of the intervention

7. Is there a follow-up or sustained evaluation of participants? Please specify when these took place.

Example: participants were evaluated 3 months after the intervention ended; intervention is cyclical, and participants are evaluated every 6 months

8. Were the recruitment strategies used reported on? If so, what was their reach?

Example: recruitment of participants by email or phone; report of the type of population that recruitment attracted

9. Were participation and dropout rates, at the various evaluation moments, assessed? If so, were they reported?

Example: 95% participation rate and 5% dropout at first assessment and 80% participation rate and 20% dropout at last assessment

10. Was an assessment of the participants' satisfaction with the intervention carried out? If so, was it reported?

Example: a questionnaire was given to participants about their satisfaction with the contents of the intervention and the performance of the practitioners involved

11. Was there any monitoring of unintended outcomes of the intervention? If so, were they reported?

Example: case of a participant who developed an eating disorder after attending healthy eating promotion sessions

12. Was there any monitoring of the consistency/fidelity or changes made to the intervention protocol? If so, were any changes made? Which ones?

Example: practitioners supervised the sessions, reporting that in a certain location they were unable to do a planned outdoor activity due to weather conditions

13. Has the magnitude of effects (effect sizes) been calculated? If so, please specify.

Effect sizes - a measure that compares the magnitude of the difference in the results obtained in different intervention groups or the magnitude of the association between two or more variables

Example: there were significant differences in the weight of participants after their involvement in the intervention

14. Was the intervention effective? That is, did the evaluation results meet the previously established intervention objectives and goals? If so, were they reported?

Example: participants increased the number of steps per day by 1000, not 1500, therefore targets were not met

15. Was there any monitoring of negative consequences and outcomes of participation in the intervention? If so, were they reported?

Example: potential risk of injury after participation in a physical activity session

Questions about implementation strategies

1. Was there training in specific aspects of implementation for the practitioners involved in the intervention?

Example: short course for the practitioners who will implement the intervention on the content of the sessions and the activities they should teach

2. What human and material resources are necessary to implement the intervention? Please specify them.

Example: use of 1 multisport center, 3 fitness balls, 3 elastic bands, 1 blood pressure machine, 1 scale, 4 practitioners and 1 nurse

3. Were previously existing resources used or integrated into the implementation?

Example: use of the exercise room of a local gym

4. Were the organizational structures clearly described and defined (i.e., workflow, tasks and responsibilities of each party involved in the intervention)?

Example: Municipal Council funds the intervention which takes place in the sports center of a school, but who organizes recruitment of participants is the local health care center

5. Were there multidisciplinary and intersectoral partnerships established for the development and implementation of the intervention? If so, which ones?

Example: universities, schools, municipalities, social institutions, private companies

6. Has a strategy been defined to keep the intervention running in the long term? If so, please describe it.

Example: existence of institutional support; support from stakeholders and partners; integration into larger scale action plans; alignment with strategic guidelines and policies

7. Was the transferability of the intervention to other populations or contexts assessed? If so, in what way will this transfer be possible?

Example: possibility of the intervention being carried out in another part of the country, taking into account political, social and economic conditions
